# Development of a Trusted Third Party at a Large University Hospital: Design and Implementation Study

**DOI:** 10.2196/53075

**Published:** 2024-04-18

**Authors:** Eric Wündisch, Peter Hufnagl, Peter Brunecker, Sophie Meier zu Ummeln, Sarah Träger, Marcus Kopp, Fabian Prasser, Joachim Weber

**Affiliations:** 1 Core Unit THS, Berlin Institute of Health at Charité – Universitätsmedizin Berlin, Berlin, Germany; 2 Digital Pathology, Charité – Universitätsmedizin Berlin, Berlin, Germany; 3 Core Unit Research IT, Berlin Institute of Health at Charité – Universitätsmedizin Berlin, Berlin, Germany; 4 Medical Informatics Group, Center of Health Data Science, Berlin Institute of Health at Charité – Universitätsmedizin Berlin, Berlin, Germany; 5 Center for Stroke Research Berlin, Charité – Universitätsmedizin Berlin, Berlin, Germany; 6 German Centre for Cardiovascular Research (DZHK), Berlin, Germany

**Keywords:** pseudonymisation, architecture, scalability, trusted third party, application, security, consent, identifying data, infrastructure, modular, software, implementation, user interface, health platform, data management, data privacy, health record, electronic health record, EHR, pseudonymization

## Abstract

**Background:**

Pseudonymization has become a best practice to securely manage the identities of patients and study participants in medical research projects and data sharing initiatives. This method offers the advantage of not requiring the direct identification of data to support various research processes while still allowing for advanced processing activities, such as data linkage. Often, pseudonymization and related functionalities are bundled in specific technical and organization units known as trusted third parties (TTPs). However, pseudonymization can significantly increase the complexity of data management and research workflows, necessitating adequate tool support. Common tasks of TTPs include supporting the secure registration and pseudonymization of patient and sample identities as well as managing consent.

**Objective:**

Despite the challenges involved, little has been published about successful architectures and functional tools for implementing TTPs in large university hospitals. The aim of this paper is to fill this research gap by describing the software architecture and tool set developed and deployed as part of a TTP established at Charité – Universitätsmedizin Berlin.

**Methods:**

The infrastructure for the TTP was designed to provide a modular structure while keeping maintenance requirements low. Basic functionalities were realized with the free MOSAIC tools. However, supporting common study processes requires implementing workflows that span different basic services, such as patient registration, followed by pseudonym generation and concluded by consent collection. To achieve this, an integration layer was developed to provide a unified Representational state transfer (REST) application programming interface (API) as a basis for more complex workflows. Based on this API, a unified graphical user interface was also implemented, providing an integrated view of information objects and workflows supported by the TTP. The API was implemented using Java and Spring Boot, while the graphical user interface was implemented in PHP and Laravel. Both services use a shared Keycloak instance as a unified management system for roles and rights.

**Results:**

By the end of 2022, the TTP has already supported more than 10 research projects since its launch in December 2019. Within these projects, more than 3000 identities were stored, more than 30,000 pseudonyms were generated, and more than 1500 consent forms were submitted. In total, more than 150 people regularly work with the software platform. By implementing the integration layer and the unified user interface, together with comprehensive roles and rights management, the effort for operating the TTP could be significantly reduced, as personnel of the supported research projects can use many functionalities independently.

**Conclusions:**

With the architecture and components described, we created a user-friendly and compliant environment for supporting research projects. We believe that the insights into the design and implementation of our TTP can help other institutions to efficiently and effectively set up corresponding structures.

## Introduction

### Background

Medical research relies on the effective collection, management, and analysis of biomedical data [1]. However, the complexity of associated data flows is increasing constantly due to the rising importance of data-driven approaches from the areas of data science and artificial intelligence [2,3]. These typically require data to be reused and shared to generate the necessary large data sets, for example in neuroscience [4]. At the same time, relevant data are often highly sensitive and require protection against unauthorized use and disclosure [5]. In alignment with this need, various laws, regulations, guidelines, and best practices suggest pseudonymization as a central data protection mechanism, especially in biomedical research [6]. Pseudonymization refers to a process in which data that directly identifies individuals (henceforth denoted as identifying data), such as names and addresses, are stored separately from data and biosamples needed for scientific analyses, and research assets are identified using protected identifiers, known as pseudonyms [7]. This protects the identity of patients or study participants while still allowing the implementation of complex research workflows, for example, data linkage. It is frequently suggested to bundle pseudonymization with other functionalities relevant to data protection and compliance, such as consent management, and that those should be carried out by particularly trusted units, knwon as trusted third parties (TTPs). One example of a concept recommending TTPs is the Guideline for Data Protection in Medical Research Projects by Technology, Methods, and Infrastructure for Networked Medical Research (TMF), the German umbrella organization for networked medical research [8].

Although the general functionalities required by medical research projects may be similar, the way they are combined into workflows often differs significantly. The reason is that due to varying study schedules and (data) modalities, studies often have different requirements concerning the necessary number and types of pseudonyms as well as the research assets that have to be registered. The timing of consent collection can also vary, for example, if reconsenting is required. Another factor that can contribute to heterogeneity is the need for integration of or linkage with data from external systems or institutions. As a result, studies often develop study- or project-specific solutions to fulfill specific registration, pseudonymization, linkage, and consenting requirements [9]. Some open tools, such as Enterprise Identifier Cross-Referencing (E-PIX) [10], Generic Pseudonym Administration Service (gPAS) [11], Generic Informed Consent Service (gICS) [12], or Mainzelliste [13], have been developed and are in widespread use; however, they are usually not integrated with each other, making the implementation of more complex workflows involving different TTP operations challenging and potentially lead to systematic limitations (explained further in the *Discussion* section). Although research exists on the components mentioned above, the literature lacks insights into the design of more comprehensive architectures that support complex research workflows that are actually in production use [14,15].

### Objectives

This paper presents the design of a comprehensive architecture for a TTP that aims to support a wide range of different research projects and studies using a unified system. As a first step, we present requirements elicited for this structure and then describe the implementation of a corresponding solution that reuses existing open components. These components are extended with a common application programming interface (API) and a common graphical user interface (GUI). We then present insights into our experiences with piloting this structure and describe our plans for future developments.

## Methods

### Requirements

TTPs typically offer a range of core functionalities based on their role in supporting research projects and clinical studies with data protection services. Three key functionalities provided are as follows: (1) identity management, through which patients and study participants are registered and their identities are managed across different systems using record linkage; (2) pseudonym management, which provides and manages pseudonyms for different research contexts and is thus critical for data protection compliance; and (3) consent management, to obtain and manage patient and participant consent for various research activities. Further components are usually included to make these core functionalities accessible. An API is necessary for the systematic retrieval of information, the implementation of complex workflows, and integration with further health care and research systems. Moreover, a well-designed GUI is necessary to enable TTP staff and study personnel to perform common tasks efficiently. An audit trail is required to ensure transparency and traceability. Furthermore, data import and export functions are necessary for transferring data from legacy systems and archiving in study-specific contexts. Finally, platform independence is an important nonfunctional requirement to support wide adoption.

A common set of tools providing these core functionalities and features (Table 1) are E-PIX [10], gPAS [11], and gICS [12], which are provided as free web-based software by the MOSAIC project from the University of Greifswald (explained in the following section). They are successfully used in a range of research projects and infrastructures [16]. Table 1 illustrates which of the above-mentioned core requirements are fulfilled by which of the MOSAIC tools.

**Table 1.** Core functional requirements and MOSAIC tools that fulfill them.

Core functional requirements		Tools		
		E-PIX^a^	gPAS^b^	gICS^c^
**Basic services**				
	Identity management	✓	—^d^	—
	Pseudonym management	—	✓	—
	Consent management	—	—	✓
**Additional features**				
	Application programming interface	✓	✓	✓
	Graphical user interface	✓	✓	✓
	Audit trail	✓	—	✓
	Data import and export	✓	✓	✓

a E-PIX: Enterprise Identifier Cross-Referencing.

b gPAS: Generic Pseudonym Administration Service.

c gICS: Generic Informed Consent Service.

d Not applicable.

Although the MOSAIC tools provide the basic functionalities needed, we elicited additional requirements from our extensive experience with supporting research projects. An overview is provided in Table 2. A detailed discussion is available in the section *Comparison With Prior Work*.

**Table 2.** Additional functional requirements and core services for which they are relevant.

Additional functional requirements		Identity management	Pseudonym management	Consent management
**Programmatic interfaces and workflows**				
	Modern REST^a^ application programming interface	✓	✓	✓
	Information exchange with other systems (eg, for ingesting consents documented in the EHR^b^ system)	✓	✓	✓
	Cross-system workflows (eg, creation of a primary identifier, combined with the creation of all necessary pseudonyms based on the domain tree and preparation of a consent document)	✓	✓	✓
**User interfaces and services**				
	Integrated user interface across all services	✓	✓	✓
	Common authentication and authorization framework with single-sign-on and associated rights and roles with the ability to connect to institutional directory services	✓	✓	✓
	Sending status messages to users in case of relevant events (eg, when a new patient has been registered)	✓	✓	✓
**Specific features**				
	Visualization of pseudonyms as QR codes	—^c^	✓	—
	Automated versioning when storing consent updates	—	—	✓
	Kiosk mode for consent documentation	—	—	✓

a REST: representational state transfer.

b EHR: electronic health record.

c Not applicable.

#### Programmatic Interfaces and Workflows

Representational state transfer (REST) services have become a de facto standard for modern applications over the last couple of years, as they are stateless, lean, and based on open web standards. Hence, we considered a REST API to be an important requirement for all 3 areas—identity management, pseudonym management, and consent management. Together with other common technologies, such asJavaScript Object Notation, this makes the services offered by the TTP accessible to other systems and processes. It also fosters effective information exchange with other systems, for example, to automatically generate primary identifiers and pseudonyms in case a patient is registered in the electronic health record (EHR) system. Moreover, a common API across all services also enables cross-service workflows, which we consider particularly important. An example of this is the automatic creation of pseudonyms linked to the primary identifier when registering a patient or study participant.

#### User Interfaces and Services

We considered an integrated user interface (UI) together with a shared authentication and authorization mechanism to be central for our TTP infrastructure. Important functionalities that the UI needs to support include depseudonymization, patient and participant registration, consent management and configuration, as well as administration. A tighter integration of the different components also facilitates sending status messages to users in case actions are required on their side.

#### Specific Features

We further identified requirements in regard to specific management functionalities. For example, representing pseudonyms as QR codes is important for seamless workflows across different media; this includes printing the codes on accompanying documents or biospecimen tubes and then reading them using QR code readers. This is particularly important for biospecimen management. Moreover, we identified a need for versioning of managed consent documents. In the event of updates to consents, for example, due to wrong information on the consent form, versioning of the various consents in the system is important for traceability. This also requires the system to be able to assign consents or withdrawals to other participants (eg, if a wrong identifier has been used when originally collecting the form). In addition, a kiosk mode that locks the user into the application is needed for the secure collection of consents from patients using tablets.

#### Nonfunctional Requirements

The most important nonfunctional requirements are as follows: (1) scalability, particularly when executing cross-service operations, and (2) documentation of administration functions.

### Building Blocks

In this section, we will describe basic building blocks of the developed application stack.

#### MOSAIC Tools

As mentioned previously, the application has been developed around the MOSAIC tools [17] as core components. Although these tools do not fulfill all our requirements, they provide a solid basis for implementing the core functionalities. The MOSAIC tools have been positively evaluated by the data protection authority of Mecklenburg-Vorpommern in Germany [18] and have been successfully used in several research projects, for example, the BeLOVE (Berlin Longterm Observation of Vascular Events) [19,20] and NAKO (German National Cohort) studies [21].

The MOSAIC suite consists of 3 tools [22]: E-PIX provides a master patient index following the Integrating the Healthcare Enterprise (IHE) profiles, Patient Identifier Cross-Reference (PIX), and Patient Demographics Query [23,24]; gPAS provides associated pseudonymization functionalities; and gICS supports integrated consent management. More specifically, E-PIX enables the central management of directly identifying master data and supports probabilistic record linkage. The resolution of potential matches between identifying data is supported through the UI. gPAS supports the generation and management of pseudonyms on top of the identities managed by E-PIX using different pseudonym domains that can refer to different systems, locations, or contexts. Finally, gICS supports digitally managing informed consent and supports different consent templates and associated use policies.

Following our requirements, we implemented an authentication and authorization model as well as programmatic interfaces and graphical UIs around E-PIX, gPAS, and gICS to enable integrated workflows across all 3 tools and to improve their interfaces.

#### Authorization and Authentication

**Figure 1.**
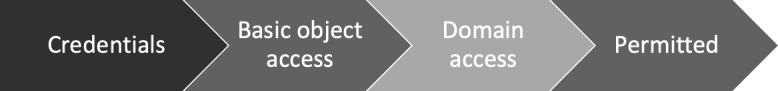
Stages of the functional authorization model.

We designed a simple, yet flexible 3-stage authorization model, which combines permissions for basic object access with permissions regarding the domain of the object to be accessed (with create, read, write, or delete permissions) by a machine or human user of the infrastructure. An overview is provided in Figure 1.

A domain defines the scope of the data managed by the TTP (eg, a research process, a study, a project, or an institute). Multiple domains can be created within a project (eg, to store pseudonyms used in specific subprojects or contexts). Additionally, in gPAS, a domain can have parent and child domains. This results in a tree structure that can be used to tailor permissions to different scopes within individual projects [25].

On the implementation side, we mapped this model to OpenID Connect (OIDC), which is based on OAuth 2.0 [26]. The JavaScript Object Notation Web Token generated in this process contains role names as attributes, which are platform independent and can also be processed on mobile devices. This is important for the additional UIs that we had to develop. As an identity and access management solution, we chose Keycloak, which is in widespread use, has a native administration interface, and is published as open-source software under the Apache License 2.0. Importantly, it can also be connected to a range of directory services usually maintained by hospitals for account and permission management.

#### Programmatic Interface

We decided to implement a REST API to extend the programmatic interfaces of E-PIX, gPAS, and gICS and support cross-tool workflows. Due to its stateless nature, this design enables the management and sharing of data across different systems, combined workflows, and calls by external components. One important application of the unified REST API is to combine participant registration with automatic consent checking in gICS, indexing the participant in E-PIX, and generating pseudonyms in gPAS. Furthermore, the REST API can easily be integrated with the developed authentication and authorization model as well as logging and audit trail functionalities. Existing interfaces of MOSAIC tools can also be integrated with the permission model by wrapping them behind REST interfaces.

#### Graphical Interfaces

##### Web Interface

Based on the integrated programmatic API that supports all services, we have also implemented an integrated GUI, which allows accessing all TTP services in a unified manner. Analogously to the programmatic API, the UIs are integrated with the described authentication and authorization model. Users can log into the platform with their account from the connected directory service, which is abstracted way using OIDC with Keycloak. The token generated at log-in contains all assigned permissions, which are used in the UI and sent as a bearer token with each request to the REST services. A strict content-security-policy workflow blocks the execution of foreign scripts outside the origin domain, thus increasing the level of security. Actions such as participant administration, depseudonymization, or consent administration can be performed through wizards. Users can request essential documents, such as copies of consent, directly from the web application.

##### Mobile App

**Figure 2.**
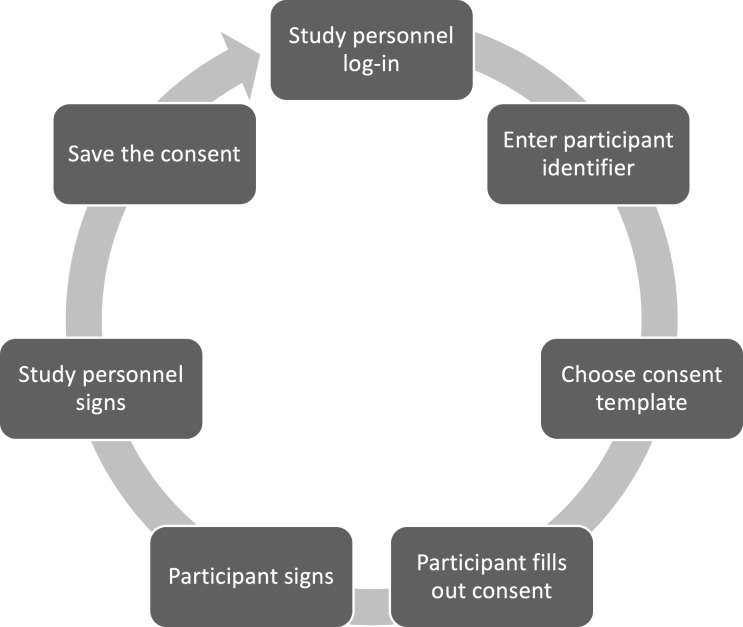
Workflow of actions in the app.

The final building block is provided by a mobile app that serves as a direct channel from the TTP services to the participants. The most important application is collecting consent and handling withdrawals. A typical deployment consists of installing the appl on a tablet, which is then configured by study personnel and handed over to the participants (Figure 2).

The study personnel can log into the app using the same log-in data as for the TTP web interface. After the project staff member enters a participant identification code and selects either a consent or a withdrawal form, the selected participant fills out the form. To prevent participants from accessing unauthorized information, the app will be started in kiosk mode. The identification code is either a temporary pseudonym or an already existing pseudonym for the participant, providing direct linkage to the research project managed by the TTP. In the latter case, the app automatically opens the associated consent template. After filling out the form, the participants can enter their name and place of residence, and then, they can put their signature in a designated field. Afterwards, the staff member provides their signature, confirming that the form has been completed with them as the assigned project staff member.

### Supported Pseudonym Algorithms

In our system, generated random numbers are used as pseudonyms. The length is configurable, with a minimum of 6 digits, and is chosen based on the number of pseudonyms that are needed for the respective project. Additionally, we use the Damm algorithm to detect single-digit errors and all adjacent transposition errors with a simple checksum [27]. Moreover, pseudonyms are combined with study- and context-specific prefixes. For example, the pseudonym “BLV-US-123456” could represent an ultrasound (“US”) measurement for a study participant in a study called BeLOVE (“BLV”). Finally, our system can also import and manage existing pseudonyms. As those are usually generated using different algorithms and often do not contain a checksum, we mark them as “external” within the system.

### Ethical Considerations

This paper covers the design and implementation of a generic research service, which requires no ethics committee approval according to local policies. However, the individual studies that use the service have to apply for ethics approval. For example, the BeLOVE study, which is described as a case study in this paper, was approved by Charité’s ethics committee (vote number EA1/066/17).

## Results

In this section, we will first describe the general architecture of our solution, then cover important implementation details, and finally report on real-world experiences with the platform.

### Architecture

**Figure 3.**
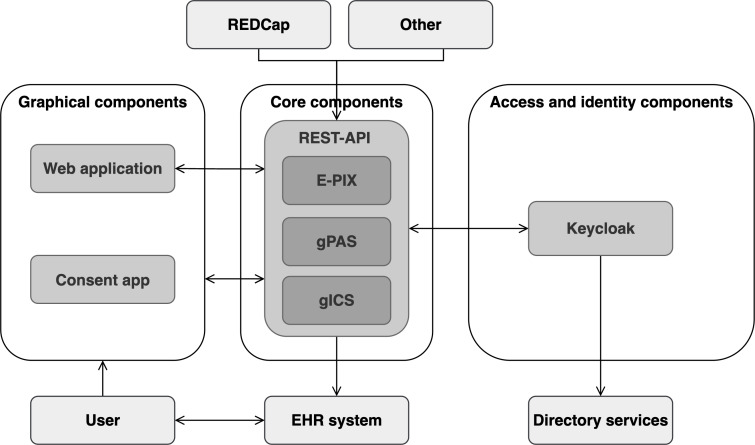
Architecture overview, including wrapped MOSAIC stack (core components); systems maintained by the trusted third party (TTP; graphical components as well as access and identity components); systems queried by the TTP (electronic health record [EHR] system and directory services); and systems from which the TTP is queried (Research Electronic Data Capture [REDCap]). E-PIX: Enterprise Identifier Cross-Referencing; gICS: Generic Informed Consent Service; gPAS: Generic Pseudonym Administration Service.

The overall architecture is divided into the API, which wraps around the MOSAIC tools, the graphical interfaces oriented toward users, as well as the access and identity management component (Figure 3 presents more details).

As illustrated, the core components are provided with an interface to the EHR system to support the pseudonymization of patient identities for direct reuse in the respective research context. Other systems that can access the TTP services via the REST API are, for example, electronic data capture systems, such as Research Electronic Data Capture (REDCap), or biobank information systems. All components of the respective interfaces are containerized with Docker [28] and deployed on a Docker swarm [29]. By using OIDC based on OAuth 2.0 as the standard, we were able to integrate other systems via existing packages (eg, Spring-Boot-Security) and allow other applications to access the systems. When modeling the interfaces, we ensured that anything that could be done graphically could also be done programmatically. This keeps the platform open and supports other information systems with the integration of TTP services.

### Implementation

**Figure 4.**
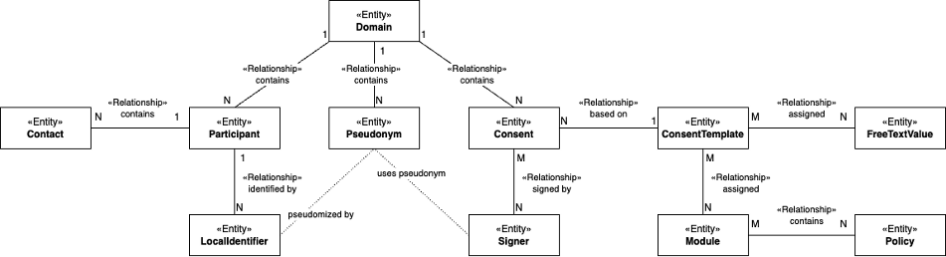
Key information objects and their relationships.

The REST API was implemented using Java 13 with the Spring Boot framework [30] by focusing on stable packages, including Spring Security for OIDC, and relying on an established framework. The resulting platform is robust, maintainable, extensible, and flexible. We have implemented 35 generic interfaces so far, most of which are Create-Read-Update-Delete (CRUD) interfaces for the key information objects Domain, Participant, Identifier, Pseudonym, Consent, and Consent Template (Figure 4), as well as additional directory and search functions for pseudonyms and consents.

**Figure 5.**
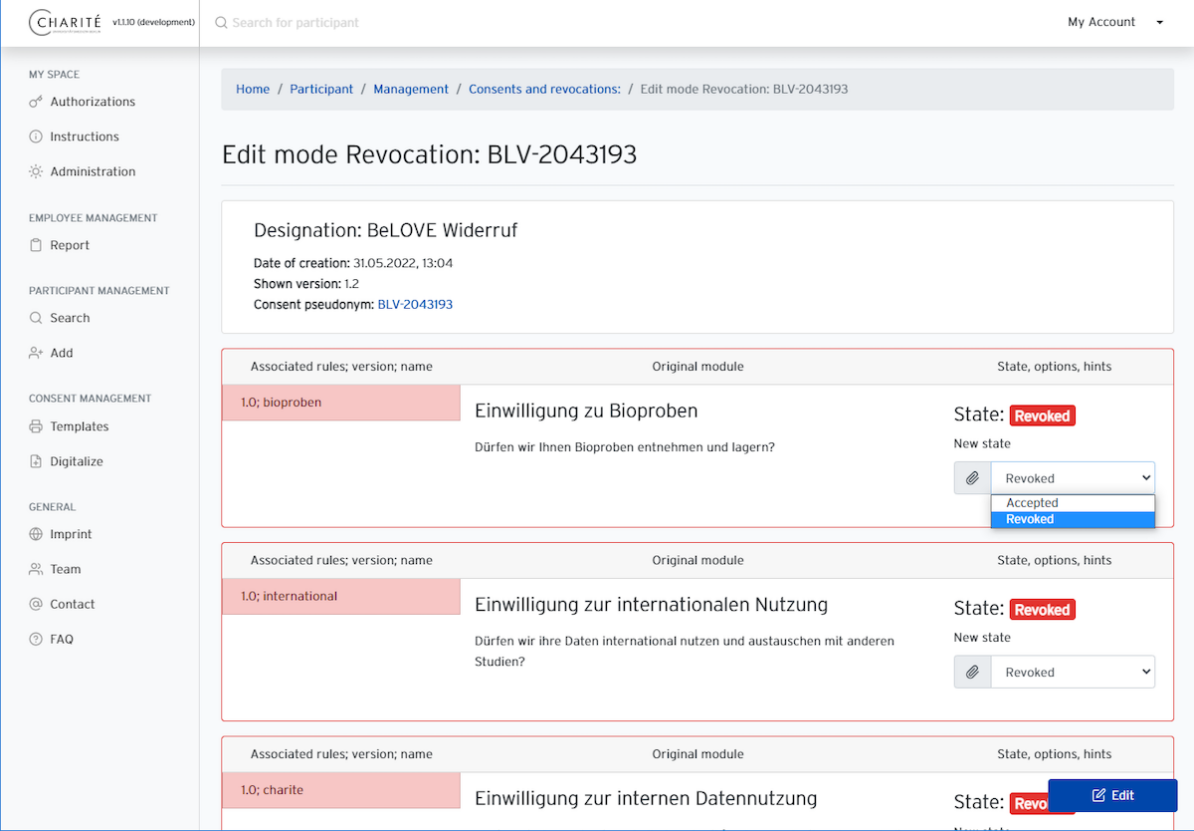
Screenshots of the user interface: editing consent information.

The web-based interface (Figures5  6) is implemented using the PHP-based lightweight enterprise web framework Laravel [31]. Laravel uses a Model-View-Controller pattern [32], has a template engine named Blade, and supports agile development processes. By integrating the open-source framework Bootstrap, we were able to implement a responsive front end that could be displayed in browsers on multiple types of devices. The web application directly interfaces with the REST API and does not manage any participant data in a separate database.

**Figure 6.**
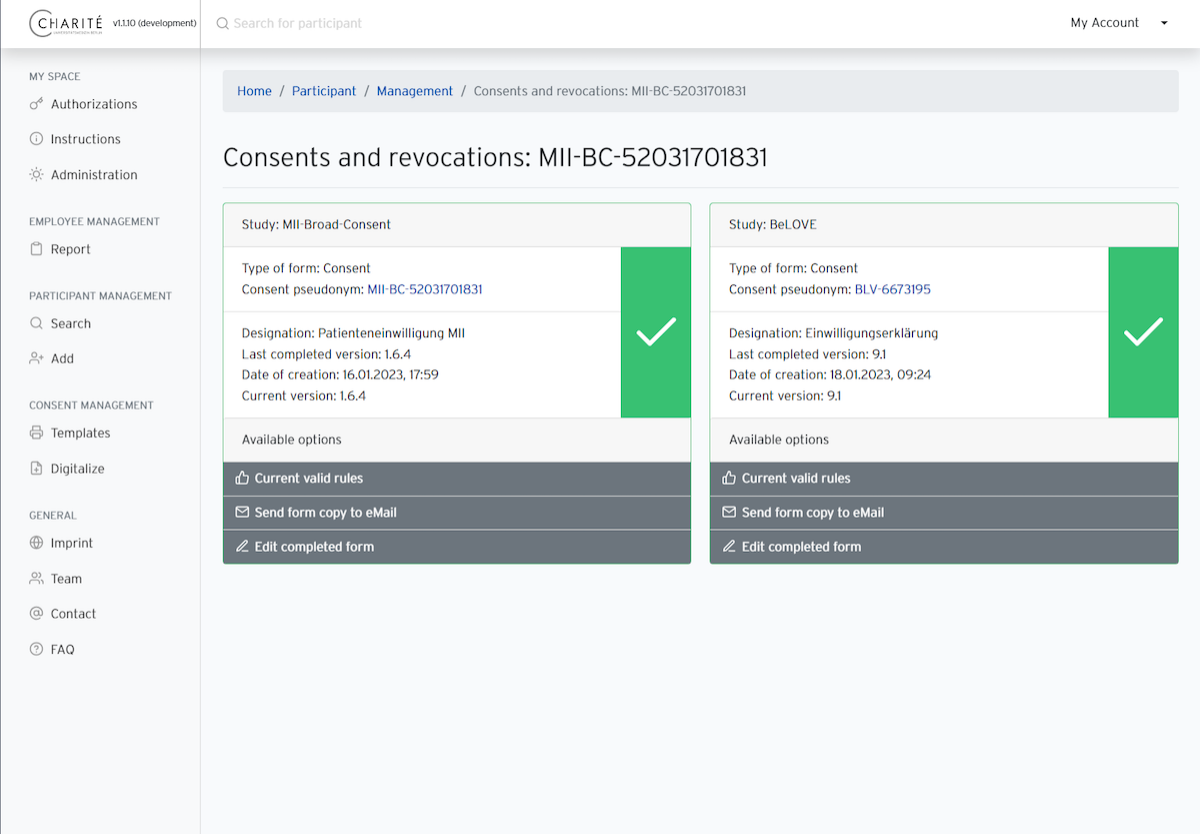
Screenshot of the user interface: overview of consent status.

**Figure 7.**
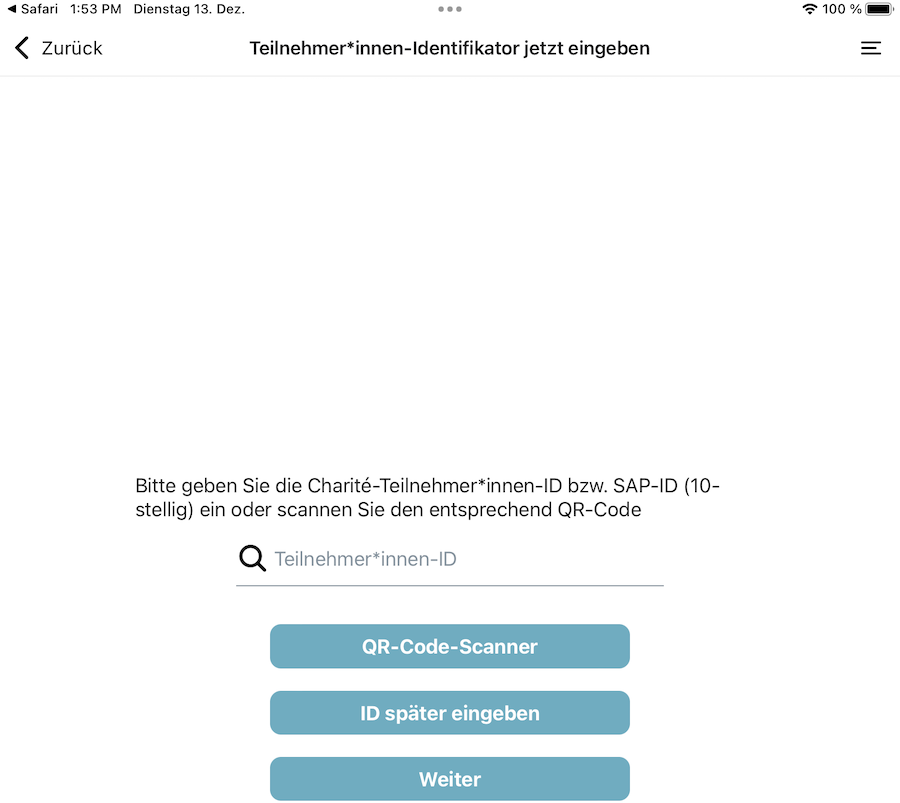
Screenshot of the consent app: entering or scanning an ID.

**Figure 8.**
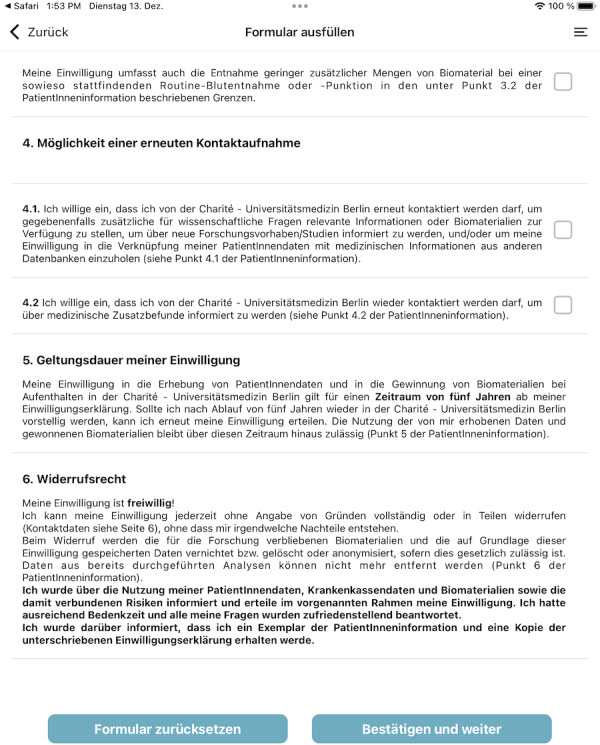
Screenshot of the consent app: filling out consent forms.

**Figure 9.**
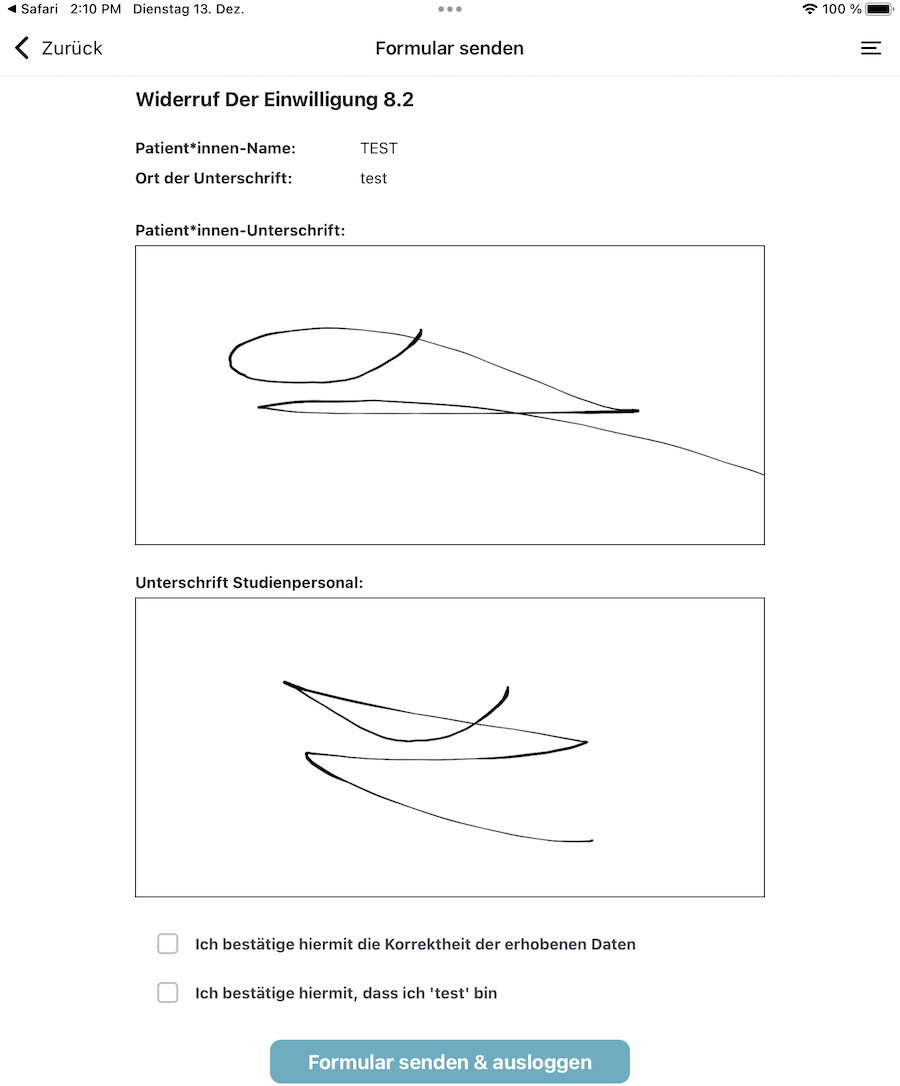
Screenshot of the consent app: sign and submit.

The app front end (see Figures 7-9) was developed in React Native [33] and then significantly extended to work on tablets integrated into our mobile device management. The application does not permanently store any data on the device, and processing is carried out exclusively via React Native state management.

### Core Functionalities for Research Projects

As a result of our development efforts, the TTP software stack provides a wide range of functionalities that research projects need. Table 3 provides an overview of frequently used common features.

**Table 3.** Essential functionalities provided to research projects.

Component	Process	Description
API^a^	Obtaining a temporary pseudonym	Automated creation of participant placeholders that can be used in third-party systems and later linked to the study identity
App	Electronic consent management	Viewing, completing, saving, and printing an electronic consent template of the respective project under a pseudonym
Web UI^b^	Participant registration	Master data and contact details can be entered manually or imported from the EHR^c^ system
Web UI	Participant overview	Provides an overview of the participants and pseudonyms associated with a specific project
API	Integration with other systems	Interface for pseudonymization, depseudonymization, and linkage for third-party systems
Web UI	Depseudonymization	Resolve pseudonym to participant master data
Web UI	Retrieval of usage permissions based on consent information	Retrieve electronic representation of usage permissions from consents associated with a specific patient or participant pseudonym
Web UI	Update participant information	Use pseudonyms to update participant information

a API: application programming interface.

b UI: user interface.

c EHR: electronic health record.

On the API level, these features include integration with other systems to manage pseudonymization, depseudonymization, and data linkage. The app specializes in electronic consent management, specifically viewing, completing, and saving of consent templates. The web-based UI permits registration of participant details; provides an overview of participants, consents, and pseudonyms; supports depseudonymization as well as the retrieval of use permissions based on consent information. CRUD operations for major participant properties and printing consents are also supported.

### Experiences in Real-World Operational Settings

The TTP has already supported more than 10 research projects since it was launched in December 2019. As of December 2022, our TTP system manages data of 3610 registered participants with 384,813 pseudonyms and 1762 consent documents. The pseudonyms fall into 2 categories: 40,867 pseudonyms have been assigned to individual participants managed by the TTP and 343,946 pseudonyms to other identifiers (eg, health insurance numbers that are managed by the TTP as part of its support for data linkage). On average, the TTP manages about 11 pseudonyms for each individual participant. As many as 153 research personnel actively engage with the software on a daily basis. Backups of our databases are created every night. These backups are stored for 90 days along with all log files.

As a case study, we will describe how the TTP services are being used by the large-scale BeLOVE study [20], which is carried out as a cooperation between several sites and departments at Charité. BeLOVE uses all services provided, from patient as well as participant registration and consent management, to pseudonym generation for the various diagnostics and phenotyping activities performed during hospitalizations or study visits (about 12 pseudonyms per participant). Compared to the initial planning of the study, which required 2 study staff for the administrative tasks, these staff requirements were in the meantime reduced to zero due to the functionality of our TTP and the associated secure outsourcing of tasks to all study staff. The use of central TTP services has also significantly reduced the efforts required for coordinating BeLOVE and its substudies with the data protection and information security officers. Within Charité’s internal data integration platform, consistent pseudonyms and API access to mapping rules are frequently used to link data collected about BeLOVE participants with routine health care data collected during inpatient and outpatient encounters for various types of analyses. Secondary pseudonyms have already been generated for 10 projects in which the data have been analyzed or shared with others.

## Discussion

### Principal Results

In this paper, we have presented a software stack to support a TTP with its core tasks at a large German academic medical center. Our architecture extends existing systems for key functionalities, identity management, pseudonymization, and consent management with a fine-grained authentication and authorization model, a modern REST API, two types of UIs, and connections to third-party systems. These extensions were necessary to support cross-service workflows on the programmatic as well as the user level and to meet further functional and nonfunctional requirements. Our application is built using various open-source enterprise frameworks and standards (eg, OIDC) to ensure sustainability and integration with important institutional services (eg, our user directory and leading master patient index). Our experiences with supporting a wide range of research projects with TTP services over a longer period have shown that our approach works and provides functionalities that are generic enough to support a wide range of applications.

### Comparison With Prior Work

Our architecture and implementation are based on the MOASIC tools [16], which we have extended with additional components to overcome functional and nonfunctional shortcomings. Most importantly, the publicly available basic versions of the MOSAIC tools are not suitable for handling more complex and flexible workflows with fine-grained authorization. For example, supporting cross-service workflows, like registering a patient, generating pseudonyms, and preparing a consent form as an integrated operation, cannot be implemented without an additional dispatcher component that is currently not publicly available. We solved this by implementing a cross-service REST API. Although the MOSAIC tools already come with an API, it is provided individually for each service and is based on the Simple Object Access Protocol [34], which originates from the IHE web service standards [35] and is complex and slow, requiring managing server-side state. Analogously to an API, the MOSAIC tools also offer GUIs. However, they are provided individually for each service and hence do not enable users to seamlessly perform operations that require interactions with multiple core services. For this reason, we developed a cross-service UI that is based on our API. Additionally, we added functionalities for generating QR codes, versioning consent documents, and starting the system in kiosk mode. Finally, our extensions also improve the system’s scalability when executing cross-service operations, such as querying for links between pseudonyms and identifiers, which can be slow when using the MOSAIC tools [36]. We also added comprehensive documentation of administration functions, which is not fully available for the current open-source versions without registration with the vendor [37].

Prior work on TTP-related services usually focused on individual components or algorithms that could support TTP operations, deployments in specific research projects, or high-level architecture overviews.

One well-known example is the one-way hash approach employed by Vanderbilt University Medical Center as part of the ingest process into their deidentified layer within a research data warehouse [38]. Pommering et al [39] describe strategies for how pseudonymization could be used in different contexts, for example, in the secondary use of EHR data or in medical research networks and biobanks. They introduced two models that support repeated depseudonymization as well as one-time use [40]. The former model was later integrated into a concept for sharing large data sets in medical research networks and biobanks [39].

Building on this, Lo Iacono [41] investigated a cryptographic approach for generating consistent pseudonyms in multicentric studies but without describing a specific implementation within a concrete project. Dangl et al [42] describe concepts and requirements for TTP services for a specific biobank of a clinical research group. Heinze et al [43] developed two services based on IHE profiles that have been implemented into the Heidelberg Personal Electronic Health Record. One service is used to capture patient consent, while the other provides a GUI to manage consents. Further components (eg, for pseudonym or identity management) were not described in detail.

Lablans et al [13] introduce the Mainzeliste, which supports managing patient identities and pseudonyms through a web-based front end. Bialke et al [10] introduce the MOSAIC tools, which we also use in our work, as a set of tools supporting central data management for studies or research networks. They also introduce the “dispatcher” as an additional component for building complex workflows [22], which is, as we described above, unfortunately not publicly available.

Aamot et al [44] compare different strategies for depseudonymization in which, among others, the strategy of Pommering et al [39] is compared with alternative approaches. Based on this comparison, they develop a pseudonymization approach using deterministic one-way mappings based on cryptographic protocols. Lautenschläger et al [45] implement and describe a generic and tightly coupled architecture and component for pseudonymization that has been used in several research projects. On the application side, Bahls et al [14] describe a TTP architecture using the MOSAIC tools for the Routine Anonymized Data for Advanced Health Services Research project. Hampf et al [17] benchmark parts of the MOSAIC tools and conclude that it would take several days to register 2 million patients with the hardware setup utilized.

### Limitations and Future Work

As the most recent versions of the MOSAIC tools are not distributed as open-source software in a public repository [37], it was not possible for us to make changes to the core tools used. Instead, workarounds had to be implemented at the API or UI level, which is not ideal from an architecture perspective. Moreover, our TTP platform is currently focused on providing intra-institutional services only. In future work, we plan to extend our platform with external interfaces, enabling the TTP to act as a central trustee for multicentric projects. We also aim to implement additional programmatic interfaces following international interoperability standards, in particular, Health Level 7 Fast Healthcare Interoperability Resources [46] and enable study personnel to directly manage the permissions of associated staff. Finally, we plan to introduce a unified pool of consent policy keys to harmonize the permission information that can be queried from our system to enable automated downstream processing that considers consent information.

### Conclusions

Scalable and comprehensive TTP services are central to modern data-driven medical research. However, community-based comprehensive platforms that can be used to implement such services are still lacking. We believe that our description of key requirements as well as the insights provided into our flexible architecture that combines core tools with user- and application-oriented workflows and interfaces, including third-party applications, can help other institutions setting up comparable services.

## Abbreviations

API: application programming interface
BeLOVE: Berlin Longterm Observation of Vascular Events
BLV: BeLOVE
CRUD: Create-Read-Update-Delete
E-PIX: Enterprise Identifier Cross-Referencing
EHR: Electronic Health Record
gICS: Generic Informed Consent Service
gPAS: Generic Pseudonym Administration Service
GUI: Graphical user interface
IHE: Integrating the Healthcare Enterprise
NAKO: German National Cohort
OIDC: OpenID Connect
PHP: Hypertext Preprocessor
PIX: Patient Identifier Cross-Reference
REDCap: Research Electronic Data Capture
REST: representational state transfer
TMF: Technology, Methods, and Infrastructure for Networked Medical Research
TTP: trusted third party

## References

[1] Pommerening K, Sax U, Müller T, Speer R, Ganslandt T, Drepper J (2008). Integrating eHealth and medical research: the TMF data protection scheme. EHealth Comb Health Telemat Telemed Biomed Eng Bioinforma Edge.

[2] Borda A, Gray K, Fu Y (2019). Research data management in health and biomedical citizen science: practices and prospects. JAMIA Open.

[3] Wang X, Williams C, Liu ZH, Croghan J (2019). Big data management challenges in health research-a literature review. Brief Bioinform.

[4] Zhao Z, Chuah JH, Lai KW (2023). Conventional machine learning and deep learning in Alzheimer's disease diagnosis using neuroimaging: a review. Front Comput Neurosci.

[5] Eggert K, Wüllner U, Antony G (2007). Data protection in biomaterial banks for Parkinson's disease research: the model of GEPARD (Gene Bank Parkinson's Disease Germany). Mov Disord.

[6] Bourka A, Drogkaris P (2019). Recommendations on Shaping Technology According to GDPR Provisions - An Overview on Data Pseudonymisation.

[7] Kohlmayer F, Lautenschläger R, Prasser F (2019). Pseudonymization for research data collection: is the juice worth the squeeze?. BMC Med Inform Decis Mak.

[8] Pommerening K, Drepper J, Helbing K, Ganslandt T (2015). Leitfaden Zum Datenschutz in Medizinischen Forschungsprojekte.

[9] Lowrance W (2003). Learning from experience: privacy and the secondary use of data in health research. J Health Serv Res Policy.

[10] Bialke M, Bahls T, Havemann C (2015). MOSAIC—a modular approach to data management in epidemiological studies. Methods Inf Med.

[11] Geidel L, Bahls T, Hoffmann W (2013). Generische Pseudonymisierung ALS Modul des Zentralen Datenmanagements Medizinischer Forschungsdaten. Universitätsmedizin.

[12] Rau H, Geidel L, Bialke M (2020). The generic informed consent service gICS: implementation and benefits of a modular consent software tool to master the challenge of electronic consent management in research. J Transl Med.

[13] Lablans M, Borg A, Ückert F (2015). A restful interface to pseudonymization services in modern web applications. BMC Med Inform Decis Mak.

[14] Bahls T, Pung J, Heinemann S (2020). Designing and piloting a generic research architecture and workflows to unlock German primary care data for secondary use. J Transl Med.

[15] Bruland P, Doods J, Brix T, Dugas M, Storck M (2018). Connecting healthcare and clinical research: workflow optimizations through seamless integration of EHR, pseudonymization services and EDC systems. Int J Med Inform.

[16] Projekte. Unabhängige Treuhandstelle.

[17] Hampf C, Geidel L, Zerbe N (2020). Assessment of scalability and performance of the record linkage tool E-PIX in managing multi-million patients in research projects at a large university hospital in Germany. J Transl Med.

[18] Unabhängige Treuhandstelle der Universitätsmedizin Greifswald. Universitätsmedizin.

[19] Siegerink B, Weber J, Ahmadi M (2019). Disease Overarching mechanisms that explain and predict outcome of patients with high cardiovascular risk: rationale and design of the Berlin long-term observation of vascular events (Belove) study. medRxiv.

[20] Weber JE, Ahmadi M, Boldt L-H (2023). Protocol of the Berlin long-term observation of vascular events (BeLOVE): a prospective cohort study with deep Phenotyping and long-term follow up of cardiovascular high-risk patients. BMJ Open.

[21] Bozoyan C, Fitzer K, Ostrzinski S (2014). Unabhängige Treuhandstelle (THS). NAKO Treuhandstellenkonzept.

[22] Bialke M, Penndorf P, Wegner T (2015). A Workflow-driven approach to integrate generic software modules in a trusted third party. J Transl Med.

[23] GmbH GG Das Sollten SIE Über EAN Nummern Wissen. GS1 Germany.

[24] 23 patient identifier cross-referencing Hl7 V3 (Pixv3). IHE International.

[25] Hampf C, Bialke M (2023). Unabhängige Treuhandstelle der Universitätsmedizin Greifswald. gPAS Anwenderhandbuch.

[26] Ma W, Sartipi K, Sharghigoorabi H, Koff D, Bak P (2016). Openid connect as a security service in cloud-based medical imaging systems. J Med Imaging (Bellingham).

[27] Damm MH (2004). Total Anti-Symmetrische Quasigruppen [article in German].

[28] (2023). Docker overview. Docker Docs.

[29] (2023). Docker swarm overview. Docker Docs.

[30] Spring Boot.

[31] The PHP framework for web artisans. Laravel.

[32] Krasner G, Pope S (1988). A cookbook for using the model-view controller user interface paradigm in Smalltalk-80. JOOP.

[33] Kopp M (2021). Entwicklung Einer App Zur Erfassung von Einverständniserklärungen Zur Datenverarbeitung Im Rahmen Einer Medizinischen Studie an Der Charité Berlin.

[34] SOAP version 1.2 part 1: messaging framework (second edition). W3.

[35] Appendix V: web services for IHE transactions. https://profiles.ihe.net/ITI/TF/Volume2/ch-V.html.

[36] Fischer H, Röhrig R, Thiemann VS (2019). A generic IT infrastructure for identity management and pseudonymization in small research projects with heterogeneous and distributed data sources under consideration of the GDPR. Stud Health Technol Inf.

[37] Community. Unabhängige Treuhandstelle.

[38] Danciu I, Cowan JD, Basford M (2014). Secondary use of clinical data: the Vanderbilt approach. J Biomed Inform.

[39] Pommerening K, Schröder M, Petrov D, Schlösser-Faßbender M, Semler SC, Drepper J (2006). Pseudonymization service and data custodians in medical research networks- and biobanks. https://dl.gi.de/handle/20.500.12116/23646.

[40] Pommerening K, Reng M (2004). Secondary use of the EHR via pseudonymisation. Stud Health Technol Inform.

[41] Lo Iacono L (2007). Multi-centric universal pseudonymisation for secondary use of the EHR. Stud Health Technol Inform.

[42] Dangl A, Demiroglu SY, Gaedcke J (2010). The IT-infrastructure of a biobank for an academic medical center. Stud Health Technol Inform.

[43] Heinze O, Birkle M, Köster L, Bergh B (2011). Architecture of a consent management suite and integration into IHE-based regional health information networks. BMC Med Inform Decis Mak.

[44] Aamot H, Kohl CD, Richter D, Knaup-Gregori P (2013). Pseudonymization of patient Identifiers for translational research. BMC Med Inform Decis Mak.

[45] Lautenschläger R, Kohlmayer F, Prasser F, Kuhn KA (2015). A generic solution for web-based management of pseudonymized data. BMC Med Inform Decis Mak.

[46] HL7 FHIR https://www.hl7.org/fhir/.

